# Association between serum chloride levels and estimated glomerular filtration rate among US adults: evidence from NHANES 1999–2018

**DOI:** 10.1007/s11255-024-04119-0

**Published:** 2024-06-19

**Authors:** Peipei Zhao, Yiping Li, Zhewei Fei, Leyi Gu, Baosan Han, Ping Ye, Huili Dai

**Affiliations:** 1 Department of Nephrology, Molecular Cell Lab for Kidney Disease, Shanghai Peritoneal Dialysis Research Center, Renji Hospital, Uremia Diagnosis and Treatment Center, Shanghai Jiao Tong University School of Medicine, Shanghai, 200127 China; 2 State Key Laboratory of Systems Medicine for Cancer, Shanghai Cancer Institute, Renji Hospital, School of Medicine, Shanghai Jiao Tong University, Shanghai, 200032 China; 3 Shanghai University of Traditional Chinese Medicine, Shanghai, 201203 China; 4 School of Health Science and Engineering, University of Shanghai for Science and Technology, Shanghai, 200093 China; 5 Chongming Branch, Xinhua Hospital, School of Medicine, Shanghai Jiao Tong University, Shanghai, 202150 China; 6 Department of Breast Surgery, Xinhua Hospital, School of Medicine, Shanghai Jiao Tong University, Shanghai, 200092 China

**Keywords:** Serum chloride, Estimated glomerular filtration rate (eGFR), Chronic kidney disease, National Health and Nutrition Examination Survey (NHANES), Epidemiology

## Abstract

**Purpose:**

Chloride, the predominant anion in extracellular fluid from humans, is essential to maintaining homeostasis. One important metric for thoroughly assessing kidney function is the estimated glomerular filtration rate (eGFR). However, the relationship between variations in serum chloride concentration and eGFR in general populations has been poorly studied. Therefore, the purpose of this study is to elucidate the correlation between serum chloride levels and eGFR within the United States’ adult population.

**Methods:**

This cohort study was conducted using data from the National Health and Nutrition Examination Survey (NHANES), which covered the years 1999–2018. We employed multiple linear regression analysis and subgroup analysis to evaluate the correlation between serum chloride concentration and eGFR. To examine the nonlinear association between serum chloride levels and eGFR, restricted cubic spline analyses were employed.

**Results:**

Data from 49,008 participants in this cohort study were used for the chloride analysis. In the comprehensively adjusted model, a noteworthy inverse relationship was discovered between chloride plasma concentration and eGFR. Restricted cubic spline analyses revealed a significant nonlinear relationship between chloride levels and eGFR (*P* for overall < 0.001 and *P* for nonlinear < 0.001). A significant interaction was observed between eGFR and plasma chloride concentration (all *P* < 0.001 for interaction) among the subgroups characterized by sex, household income to poverty ratio, BMI, hypertension, and diabetes.

**Conclusion:**

Our findings suggest that higher levels of chloride plasma concentration were linked to decreased eGFR. These findings underscore the significance of monitoring chloride plasma concentration as a potential indicator for identifying individuals at risk of developing chronic kidney disease (CKD).

## Introduction

Chronic kidney disease (CKD) is acknowledged as a major worldwide public health issue [1, 2]. About 15–20% of the global population in the US suffer from CKD [1, 3], which is characterized by the presence of albuminuria (urine albumin to creatinine ratio [UACR] ≥ 30 mg/g) or an estimated glomerular filtration rate (eGFR) below 60 mL/min/1.73 m^2^ persisting for at least 3 months [4, 5]. Increased incidences of kidney failure necessitating kidney replacement therapy, acute renal damage, overall mortality, and cardiovascular-related mortality have all been linked to reduced eGFR levels and intensified albuminuria. A study from the Global Burden of Disease group, published in 2020, identified CKD as a leading factor among the top ten in global poor prognosis [6]. The eGFR is commonly considered the most precise measure for assessing kidney function. In standard practice, serum creatinine serves as the predominant biochemical parameter for eGFR [7]. CKD is a prevalent disorder marked by the progressive decline in renal function, which is characterized by intimal fibrosis, glomerulosclerosis and tubule-interstitial alterations (including renal tubular atrophy and fibrosis), ultimately destroying kidney parenchyma and renal failure [8]. This progression may result in a gradual decrease in eGFR [9]. The eGFR plays a crucial role in categorizing stages and risks for CKD patients, given that a reduced eGFR is independently and progressively linked to a heightened risk of end-stage kidney disease (ESKD) and death [10–12]. eGFR can be calculated from routinely collected variables such as age, race, sex, and serum creatinine [13]. Studies have shown eGFR to be a strong indicator of various outcomes, including cardiovascular events, early mortality, increased hospitalizations, and kidney failure necessitating renal replacement therapy [14, 15]. A comprehensive understanding of eGFR allows clinicians to prescribe appropriate drugs, liquid remedies, and early interventions to avert end-stage renal failure [16].

Chloride, as the primary potent anion in human extracellular fluid, makes up about 33% of the plasma’s tonicity and 67% of its total negative charges. Their main restriction is to the fluid compartment outside the cell [17–19]. Chloride in the blood plays a vital role in a variety of physiological functions in the human body, including regulating osmotic pressure, maintaining the electrical balance of bodily fluids, facilitating muscle function, and controlling blood pressure [18, 20, 21]. The kidney and gastrointestinal system are primarily responsible for controlling serum chloride levels. Chloride, excreted in the form of hydrochloric acid within gastric juice, may be absorbed throughout the entirety of the intestinal tract during digestion. American adult male chloride consumption varies between 7.8 and 11.8 g daily, compared to 5.8–7.8 g for adult female intake. Most human body chloride comes from table salt (Na Cl) in dietary intake, as well as from foods that are rich in salt [20]. The kidney is the main organ that excretes chloride. Approximately, 19,440 millimoles (mmol) are filtered daily by the kidneys, with a significant 99.1% being reabsorbed, resulting in a mere 180 mmol being expelled daily. Most reabsorption is carried out in the proximal tubule by active coupled transport with other ions, passive reabsorption, or ion conductance [17, 20]. Serum chloride levels are often overlooked in laboratory testing and are usually only taken into account in cases of metabolic acidosis. Chloride, however, has emerged as a key player in a number of homeostatic processes, such as blood pressure response, renal sodium management, tubuloglomerular feedback, and renin secretion regulation [22–26]. A growing quantity of studies are recognizing chloride’s importance, leading to its escalated utility in therapy for forecasting adverse results. In those suffering from heart failure, lower chloride levels in their serum are associated with elevated plasma renin, reduced effectiveness of diuretics, and a slower rate of muscle clearance [27–29]. Previous studies indicate a connection between hypochloremia and an increased mortality risk in individuals with pulmonary arterial hypertension (PAH), independent of their serum sodium concentrations [30, 31]. In addition, among hypertension patients, a correlation between Cl and mortality risk was identified. After adjusting for initial confounders and the concentrations of Na+, K+, and HCO3− , a decrease of 1.5% in overall mortality correlated with a 1 mEq/L rise in serum Cl−  [32]. Consequently, regardless of sodium presence, chloride might play a important role in the physiological pathology of heart-related disorders.

Cardiovascular disease and a heightened risk of mortality are directly linked to CKD. However, there are little data on the connection between Cl and the prognosis of CKD patients. The predictive significance of Cl in patients with CKD may differ from that of other patient populations due to the frequent acid–base imbalances that impact blood Cl levels. Some prior investigations have concentrated on the correlation between chloride plasma concentration and acute kidney injury (AKI). However, they were demonstrated to have the opposite connection. A multicenter observational study that included 4234 critically ill AKI patients uncovered a link between reduced serum chloride levels and an increase in mortality in ICU and during hospitalization [33]. In marked contrast, severe hyperchloremia poses a significant risk to patient outcomes. An elevated risk of AKI is linked to a greater fluctuation in chloride levels starting from the moment of hospitalization. Moreover, severe hyperchloremia serves as an independent indicator for in-hospital acute kidney injuries and death rates [34–36]. Among chronic kidney disease patients not undergoing dialysis, having low serum chloride levels correlated with increased death rates and an elevated risk of cardiovascular incidents [37, 38]. To our knowledge, not much research has been done on the general US population about the association between variations in eGFR and chloride plasma levels. Our hypothesis was tested by conducting a cross-sectional study with 49,008 participants from the National Health and Nutrition Examination Survey (NHANES: 1999–2018) to determine if changes in blood chloride levels correlate with a heightened CKD risk in adult US citizens.

## Methods

### Study population

The NHANES, developed by the National Center for Health Statistics, serves as a program for evaluating the frequency, disease risk elements, and the physical and nutritional state of adults and kids in the United States. NHANES serves as a cross-sectional survey crafted to depict the non-institutionalized civilian demographic of America. All data are accessible to the public and can be obtained from the NHANES at https: //www.cdc.gov/nchs/nhanes/index.htm. It was mandatory for NHANES participants to provide their informed consent in writing. The National Center for Health Statistics’ Research Ethics Review Board granted it authorization. Due to the deidentified and publicly available nature of NHANES data, this research did not require institutional review board permission, as per the Common Rule. Therefore, this study was not subject to any additional ethical approvals [39].

**Fig. 1:**
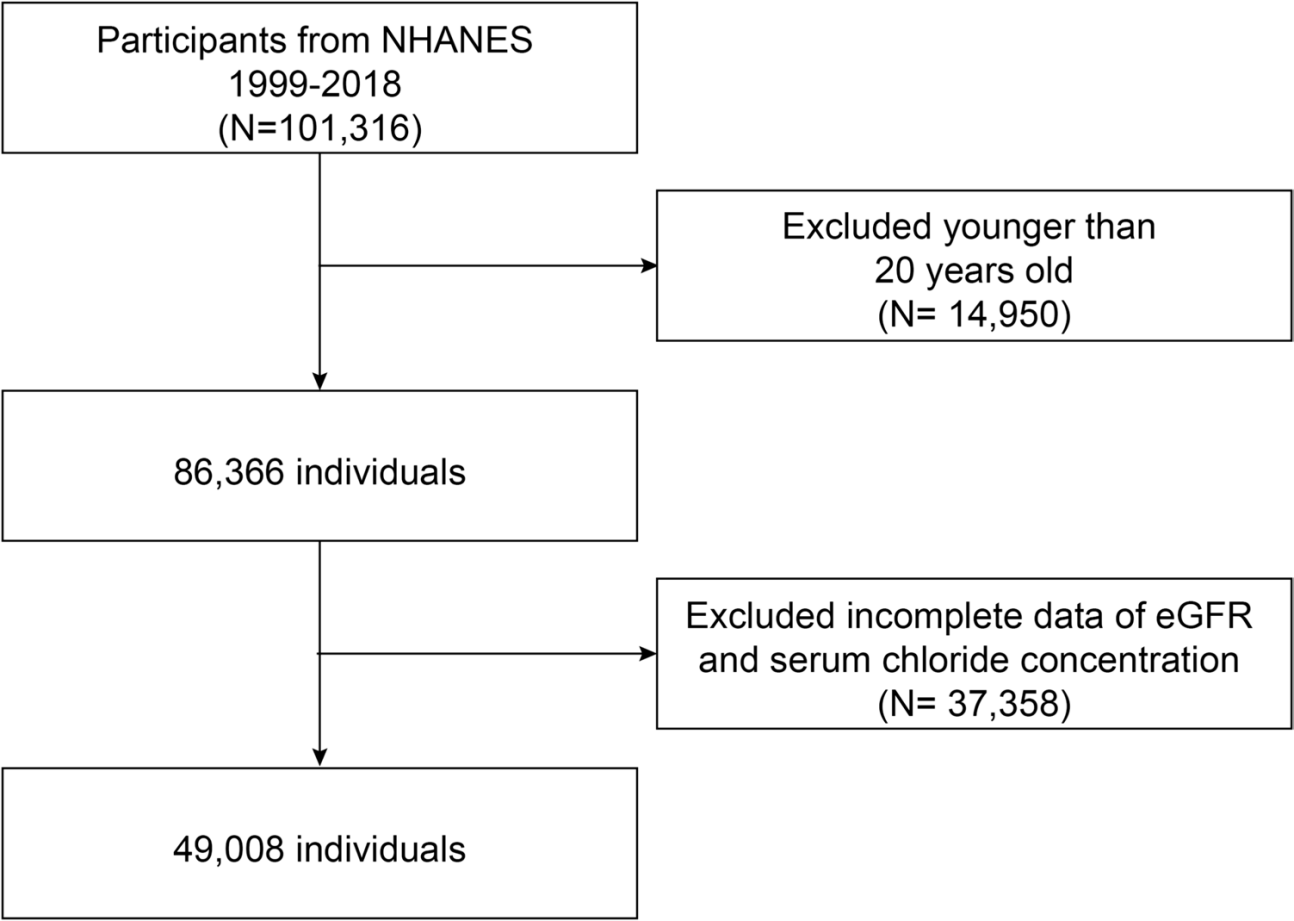
Flowchart of the screening process for selecting eligible participants. *NHANES* the National Health and Nutrition Examination Survey, *eGFR* estimated glomerular filtration rate

An analysis of ten 2-year cycles of NHANES data from 1999 to 2018 was conducted for this study. There were 101,316 individuals sampled in this study, with 86, 366 adults (age ≥ 20 years old) participants. Individuals were excluded if they lacked data on serum chloride concentration, or were missing data for the calculation of eGFR [13] (*n* = 37,358). Eventually, the present research incorporated a total of 49,008 individuals. Figure 1 displays the inclusion and exclusion criteria for the study population.

### Assessments of serum chloride concentration

Specialists with certification collect and examine blood samples at a Mobile Examination Center (MEC), from which they are subsequently preserved in biological archives. The analysis of serum chloride content was performed using Beckman Synchron LX20, Beckman UniCel DxC800 Synchron (Beckman Coulter, Fullerton, CA, USA), or Roche Cobas 6000 Chemistry Analyzer (Roche Diagnostics Corporation, Indianapolis, IN 46250). Serum chloride concentration were assessed by the ion-selective electrode indirect (or diluted) method. Classification of the individuals was based on their serum chloride concentration quartiles. All the detection procedures about serum chloride measurements have been described in detail on the NHANES websites [39].

### Measurement of estimated glomerular filtration rate

A Jaffe rate reaction was used to quantify creatinine concentration during NHANES mobile examination center screenings. Calculation of the eGFR value was based on age (in years), gender, and creatinine plasma levels (in mg/dL), utilizing the Chronic Kidney Disease Epidemiology Collaboration (CKD-EPI) equation[13].eGFR_CKD−EPI_ (mL/min/1.73 m^2^) = 141 × min (Scr/κ, 1) ^α^ × max (Scr/κ, 1) ^−1.209^ × 0.993^Age^ × 1.018 [if female] × 1.159 [if black], where Scr represents serum creatinine, κ is 0.7 for females and 0.9 for males, α is −0.329 for females and −0.411 for males, min indicates the minimum of Scr/κ or 1, and max denotes the maximum of Scr/κ or 1. The eGFR-associated data of participants were obtained from NHANES [39].

### Assessments of covariates

Following an extensive review of existing studies and our practical clinical experiences, our demographic covariates of interest included age, gender, racial and ethnicity, marital status (whether married, widowed, divorced, separated, unmarried, and cohabitating), education attainment (below 9th grade, 9 th to 11th grade, high school or GED level or something similar, college or AA education, and having a college degree or higher), and family income to poverty ratio. Based on self-report during the interview, race, and ethnicity were evaluated as fundamental demographic characteristics and categorized into five categories: Mexican American, non-Hispanic White, non-Hispanic Black, other Hispanic, and other (which includes non-Hispanic multiple races and other non-Hispanic non-Hispanic). These categories were gathered since their combined presence was thought to be confounding. We also incorporated various anthropometric measures and chronic health conditions in our study. These included body mass index (BMI), smoking habits (classified as every day, occasional, or never), alcohol consumption, hypertension (defined as being diagnosed by a physician, possessing a systolic and diastolic blood pressure levels of at least 130 mm Hg or 80 mm Hg, respectively), diabetes (self-reported diagnosis by a physician), congestive heart failure (self-reported diagnosis by a physician), stroke (self-reported diagnosis by a physician), and serum levels of total cholesterol, phosphorus, potassium, total calcium, sodium, and bicarbonate. Table 1 provides detailed information on the other categorical variables.

**Table 1:** Baseline characteristics of selected participants from the National Health and Nutrition Examination Survey (NHANES) 1999 to 2018 according to quartiles of different serum chloride concentration

Characteristic	Participants, no. (%)					*P* value
		Serum chloride levels, mmol/L				
		Quartile 1	Quartile 2	Quartile 3	Quartile 4	
	Total	70–101.3	101.4–103.4	103.5–105	105.1–120	
	(*n* = 49,008)	(*n* = 12,283)	(*n* = 12,242)	(*n* = 13,307)	(*n* = 11,176)	
Age, mean (95% CI), years	47.1 (46.8, 47.5)	49.8 (49.2, 50.3)	46.3 (45.8, 46.8)	46.1 (45.7, 46.6)	46.1 (45.6, 46.6)	<0.0001
Gender (%)						
Male	48.2 (47.8, 48.6)	53.1 (51.9, 54.2)	51.1 (50.0, 52.2)	46.0 (44.9, 47.1)	41.9 (40.8, 42.9)	<0.0001
Female	51.8 (51.4, 52.2)	46.9 (45.8, 48.1)	48.9 (47.8, 50.0)	54.0 (52.9, 55.1)	58.1 (57.1, 59.2)	
Race and ethnicity (%)						
Mexican American	8.2 (7.2, 9.4)	6.9 (5.9, 8.1)	8.0 (7.0, 9.1)	8.8 (7.7, 10.1)	9.3 (7.9, 11.0)	<0.0001
Non-Hispanic White	68.6 (66.6, 70.6)	70.3 (67.8, 72.7)	69.2 (67.0, 71.3)	68.6 (66.2, 70.9)	66.0 (63.1, 68.8)	
Non-Hispanic Black	10.7 (9.6, 1.8)	9.6 (8.4, 11.0)	10.0 (8.9, 11.1)	10.7 (9.5, 12.1)	12.6 (11.1, 14.2)	
Other Hispanic	5.6 (4.8, 6.6)	5.9 (4.8, 7.3)	5.7 (4.7, 7.0)	5.5 (4.7, 6.4)	5.4 (4.5, 6.4)	
Other race	6.9 (6.3, 7.5)	7.3 (6.3, 8.4)	7.1 (6.4, 7.9)	6.4 (5.6, 7.2)	6.7 (5.9, 7.6)	
Marital status (%)						
Married	55.9 (54.8, 56.9)	53.9 (52.4, 55.5)	56.2 (54.6, 57.7)	57.7 (56.2, 59.1)	55.5 (53.8, 57.2)	<0.0001
Widowed	6.0 (5.7, 6.3)	7.9 (7.3, 8.6)	5.5 (5.1, 6.0)	5.1 (4.7, 5.5)	5.4 (4.8, 5.9)	
Divorced	9.9 (9.5, 10.3)	10.8 (10.0, 11.6)	9.2 (8.6, 9.9)	9.4 (8.7, 10.1)	10.2 (9.5, 10.9)	
Separated	2.5 (2.3, 2.7)	2.5 (2.2, 2.9)	2.4 (2.1, 2.7)	2.5 (2.1, 2.8)	2.5 (2.2, 2.9)	
Never married	17.3 (16.5, 18.2)	17.4 (16.3, 18.5)	18.0 (16.9, 19.2)	16.8 (15.7, 18.0)	16.9 (15.7, 18.1)	
Living with partner	7.4 (7.0, 7.9)	6.3 (5.6, 7.0)	7.6 (6.8, 8.4)	7.9 (7.3, 8.7)	8.1 (7.3, 8.9)	
Missing	1.1 (0.6, 1.9)	1.2 (0.6, 2.4)	1.1 (0.6, 2.1)	0.6 (0.4, 1.0)	1.5 (0.8, 2.7)	
Educational level (%)						
Less than 9th grade	6.0 (5.6, 6.5)	6.0 (5.4, 6.7)	5.9 (5.4, 6.4)	5.7 (5.2, 6.2)	6.6 (5.9, 7.3)	0.0006
9–11th grade	11.3 (10.7, 11.9)	11.9 (11.0, 12.9)	10.5 (9.7, 11.3)	11.0 (10.2, 11.8)	12.0 (11.1, 12.9)	
High school grad/GED or equivalent	23.9 (23.2, 24.7)	24.8 (23.4, 26.2)	23.8 (22.5, 25.1)	23.3 (22.2, 24.5)	23.8 (22.6, 25.0)	
Some college or AA degree	30.9 (30.1, 31.6)	29.9 (28.6, 31.2)	30.7 (29.4, 32.1)	31.1 (29.9, 32.3)	32.0 (30.6, 33.4)	
College graduate or above	27.8 (26.4, 29.2)	27.3 (25.4, 29.4)	29.1 (27.2, 31.0)	28.8 (27.2, 30.5)	25.6 (23.7, 27.5)	
Missing	0.1 (0.1, 0.1)	0.1 (0.1, 0.2)	0.1 (0.1, 0.2)	0.1 (0.0, 0.2)	0.1 (0.1, 0.2)	
Family income to poverty ratio, mean(95% CI)	3.0 (2.9, 3.0)	3.0 (2.9, 3.0)	3.0 (3.0, 3.1)	3.0 (2.9, 3.1)	2.9 (2.8, 3.0)	<0.0001
Body Mass Index, mean(95% CI), kg/m^2^	28.8 (28.6, 28.9)	28.7 (28.4, 28.9)	28.4 (28.2, 28.6)	28.6 (28.5, 28.8)	29.4 (29.2, 29.6)	<0.0001
Smoking status (%)						
Every day	17.7 (17.0, 18.4)	17.6 (16.5, 18.7)	16.6 (15.7, 17.7)	17.3 (16.2, 18.3)	19.5 (18.3, 20.8)	<0.0001
Some days	3.7 (3.5, 3.9)	3.7 (3.3, 4.1)	3.6 (3.2, 4.0)	3.6 (3.3, 4.0)	4.0 (3.5, 4.5)	
Not at all	24.9 (24.2, 25.6)	26.4 (25.3, 27.5)	25.3 (23.9, 26.6)	24.0 (23.1, 24.9)	23.7 (22.4, 25.1)	
Missing	53.8 (52.8, 54.7)	52.3 (50.9, 53.8)	54.5 (53.0, 56.0)	55.1 (54.0, 56.2)	52.8 (51.3, 54.3)	
Had at least 12 alcohol drinks/lifetime (%)						
Yes	10.8 (10.3, 11.3)	9.5 (8.7, 10.2)	10.0 (9.4, 10.8)	11.8 (11.0, 12.7)	11.9 (11.0, 12.9)	<0.0001
No	9.7 (8.9, 10.6)	8.2 (7.4, 9.2)	9.5 (8.3, 10.8)	10.1 (9.3, 11.1)	11.2 (9.9, 12.6)	
Missing	79.5 (78.4, 80.6)	82.3 (81.0, 83.5)	80.5 (78.9, 82.0)	78.0 (76.7, 79.2)	76.9 (75.1, 78.6)	
Hypertension (%)						
Yes	49.1 (48.3, 49.9)	58.9 (57.4, 60.3)	47.9 (46.6, 49.1)	45.0 (43.7, 46.3)	44.3 (42.9, 45.7)	<0.0001
No	50.9 (50.1, 51.7)	41.1 (39.7, 42.5)	52.1 (50.9, 53.4)	55.0 (53.7, 56.3)	55.7 (54.3, 57.1)	
Missing	0.0 (0.0, 0.0)	0.0 (0.0, 0.1)	0.0 (0.0, 0.1)	0.0 (0.0, 0.0)	0.0 (0.0, 0.0)	
Diabetes (%)						
Yes	8.8 (8.5, 9.2)	13.9 (13.0, 14.7)	8.0 (7.4, 8.7)	6.6 (6.1, 7.2)	6.6 (6.1, 7.2)	<0.0001
Borderline	1.8 (1.7, 2.0)	2.0 (1.7, 2.4)	1.8 (1.5, 2.1)	1.7 (1.4, 2.0)	1.8 (1.5, 2.2)	
No	89.3 (88.9, 89.7)	84.1 (83.2, 84.9)	90.2 (89.5, 90.8)	91.6 (90.9, 92.2)	91.5 (90.8, 92.2)	
Missing	0.1 (0.0, 0.1)	0.0 (0.0, 0.1)	0.0 (0.0, 0.1)	0.1 (0.0, 0.1)	0.1 (0.0, 0.2)	
Congestive heart failure (%)						
Yes	2.4 (2.2, 2.6)	3.7 (3.3, 4.1)	2.0 (1.7, 2.3)	1.7 (1.5, 2.0)	2.1 (1.8, 2.4)	<0.0001
No	97.5 (97.3, 97.6)	96.1 (95.7, 96.5)	97.9 (97.6, 98.1)	98.2 (97.9, 98.4)	97.7 (97.4, 98.0)	
Missing	0.2 (0.1, 0.2)	0.2 (0.1, 0.3)	0.2 (0.1, 0.2)	0.1 (0.1, 0.2)	0.2 (0.1, 0.3)	
Stroke (%)						
Yes	2.8 (2.6, 3.0)	3.4 (3.0, 3.8)	2.5 (2.2, 2.9)	2.5 (2.2, 2.8)	2.7 (2.4, 3.1)	<0.0001
No	97.1 (96.9, 97.3)	96.4 (96.0, 96.8)	97.4 (97.1, 97.7)	97.5 (97.1, 97.7)	97.1 (96.8, 97.5)	
Missing	0.1 (0.1, 0.1)	0.2 (0.1, 0.3)	0.0 (0.0, 0.1)	0.0 (0.0, 0.1)	0.2 (0.1, 0.3)	
Total cholesterol, mean (95% CI), mmol/L	5.1 (5.1, 5.1)	5.2 (5.2, 5.3)	5.1 (5.1, 5.2)	5.0 (5.0, 5.1)	4.9 (4.9, 4.9)	<0.0001
Serum chloride, mean (95% CI), mmol/L	103.2 (103.1, 103.4)	99.4 (99.4, 99.5)	102.5 (102.5, 102.5)	104.5 (104.4, 104.5)	107.0 (107.0, 107.0)	<0.0001
Serum phosphorus , mean (95% CI), mmol/L	1.2 (1.2, 1.2)	1.2 (1.2, 1.2)	1.2 (1.2, 1.2)	1.2 (1.2, 1.2)	1.2 (1.2, 1.2)	<0.0001
Serum potassium, mean (95% CI), mmol/L	4.0 (4.0, 4.0)	4.0 (4.0, 4.0)	4.0 (4.0, 4.0)	4.0 (4.0, 4.0)	4.0 (4.0, 4.0)	0.0134
Serum total calcium, mean (95% CI), mmol/L	2.4 (2.4, 2.4)	2.4 (2.4, 2.4)	2.4 (2.4, 2.4)	2.4 (2.3, 2.4)	2.3 (2.3, 2.3)	<0.0001
Serum sodium, mean (95% CI), mmol/L	139.2 (139.1, 139.4)	137.8 (137.6, 138.0)	139.1 (139.0, 139.2)	139.7 (139.6, 139.8)	140.5 (140.4, 140.6)	<0.0001
Serum bicarbonate, mean (95% CI), mmol/L	24.8 (24.7, 24.9)	25.5 (25.4, 25.6)	25.1 (25.0, 25.2)	24.7 (24.6, 24.8)	23.8 (23.7, 23.9)	<0.0001

For continuous variables: survey-weighted mean (95% CI), *P* value was by survey-weighted linear regression

For categorical variables: survey-weighted percentage (95% CI), *P* value was by survey-weighted Chi-square test

### Statistical analysis

All the analyses in this paper take into account sample weights, strata, and primary sampling units because of the intricate sampling architecture of NHANES. According to the serum chloride concentration quartile, we separated the study population into four groups: 70 ≤ quartile 1 ≤ 101.3 mmol/L, 101.3 < quartile 2 ≤ 103.4 mmol/L, 103.4 < quartile 3 ≤ 105.0 mmol/L, and 105 < quartile 4 ≤ 120 mmol/L. Multiple linear regression analysis was employed to investigate the relationships between eGFR and blood chloride levels. To evaluate the study population’s initial characteristics, descriptive statistics were employed. The survey-weighted percentage with a 95% confidence range was computed for categorical variables, and the *P* value was derived via the survey-weighted Chi-square test. Regarding continuous variables, a survey-weighted average (with a 95% Confidence Interval) was computed, and in response, the *P* value derived through survey-weighted linear regression.

In addition to the above methods, we also employed weighted linear regression. When conducting regression analysis, we utilized the sample weight variables provided by the NHANES. This approach fully considered the complexity of the sample design, thereby ensuring the representativeness and accuracy of the research results. Three models were built. There was no covariate adjustment in Model 1. Age, gender, race, marital status, degree of education, and family income to poverty ratio were all taken into account in Model 2. In addition, Model 3 made adjustments for variables such as body mass index, smoking status, alcohol drinks, hypertension, diabetes, congestive heart failure, stroke, total cholesterol and serum bicarbonate. The multiple imputation using chained equations approach was used to impute covariates that had missing values. Multiple interpolation techniques were employed for continuous variables, while missing categorical variables were categorized separately. The nonlinear relationship between chloride plasm levels and eGFR was determined using multivariable restricted cubic spline (RCS) analysis. Age, gender, educational attainment, the ratio of family income to poverty, body mass index, hypertension, and diabetes were further stratified in our analyses. Age was categorized into three groups: those aged 39 or younger, those between 40 and 59, and individuals 60 years or older. Three categories were established for the family income to poverty ratio: lower than 1.0, between 1.0 and 3.0, and exceeding 3.0. BMI was determined by dividing weight in kilograms by the squared height in meters, assigning categories of less than 25.0, 25.0 to 29.9, and 30.0 or more.

The research utilized R software, version 4.3.1 (R Project for Statistical Computing), and EmpowerStats, version 6.0 for all statistical analyses conducted between October 1, 2023, and March 1, 2024. A significance criterion of *P* < 0.05 was applied to all studies.

## Results

### Baseline characteristics of the study participants

The final analysis included the 49,008 US adults qualified individuals who remained. The average age among the 49,008 individuals was 47.1 (95% CI, 46.8, 47.5) years, 51.8% of whom were female, and 68.6% of the participants were non-Hispanic White. Based on the quartiles, the participants’ serum chloride values were divided into four groups: 70 mmol/L ≤ quartile 1 ≤ 101.3 mmol/L, 101.3 mmol/L < quartile 2 ≤ 103.4 mmol/L, 103.4 mmol/L < quartile 3 ≤ 105.0 mmol/L, and 105 mmol/L < quartile 4 ≤ 120 mmol/L. After dividing participants by serum chlorideconcentration, there were 12,283 participants (mean [95% CI] age, 49.8 [49.2, 50.3] years; 6,522 [53.1%] men) in quartile 1, the lowest concentration quartile; 12,242 participants (mean [95% CI] age, 46.3 [45.8, 46.8] years; 6, 255 [51.1%] men) in quartile 2; 13,307 participants (mean [95% CI] age, 46.1 [45.7, 46.6] years; 6,121 [46.0%] men) in quartile 3; and 11,176 participants (mean [95% CI] age, 46.1 [45.6, 46.6] years; 4,682 [41.9%] men) in quartile 4, the highest concentration quartile. In summary, compared with individuals in the lowest quartile of serum chloride concentration, those with higher serum chloride were more likely to be younger, female, non-Hispanic White, married, and to drink more; they also had lower total cholesterol, lower serum total calcium, lower serum bicarbonate, and higher serum sodium levels; additionally, they were more unlikely to have hypertension, diabetes, congestive heart failure, and stroke (Table 1).

### Association between serum chloride concentration and eGFR in NHANES (1999–2018)

The associations of chloride plasma concentration and eGFR were evaluated with multiple linear regression analysis. In Model 1, the β of eGFR in the fourth quartile was 2.3 (95% CI, 1.0, 3.5) compared with the reference group (*P* < 0.001 for trend). Compared with the lowest quartile of serum chloride, the highest quartile was associated with a lower eGFR in the partly adjusted Model 2 (β = −1.9 [95% CI −2.8, −1.1]; *P* < 0.001 for trend). Following adjustments for multiple variables in Model 3, compared with the reference group (the first quartile), the β for eGFR was −1.7 (95% CI −2.3, −1.1, *P* < 0.001) in the second quartile, − 1.8 (95% CI −2.4, −1.2, *P* < 0.001) in the third quartile, and − 2.3 (95% CI −3.2, −1.4, *P* < 0.001) in the fourth quartile (*P* < 0.001 for trend) (Table 2).

**Table 2:** Associations between serum chloride concentration and eGFR

	Chloride plasma concentration, mmol/L							
	Quartile 1 (70–101.3)	Quartile 2 (101.4–103.4)		Quartile 3 (103.5–105)		Quartile 4 (105.1–120)		*P* value for trend
Model	β (95% CI^a^)	β (95% CI^a^)	*P* value	β (95% CI^a^)	*P* value	β (95% CI^a^)	*P* value	
Model 1	Reference	1.80 (1.00, 2.60)	< 0.001	2.20 (1.30, 3.10)	< 0.001	2.30 (1.00, 3.50)	< 0.001	< 0.001
Model 2	Reference	−1.50 (−2.00, −0.89)	< 0.001	−1.50 (−2.10, −0.92)	< 0.001	−1.90 (−2.80, −1.10)	< 0.001	< 0.001
Model 3	Reference	−1.70 (−2.30, −1.10)	< 0.001	−1.80 (−2.40, −1.20)	< 0.001	−2.30 (−3.20, −1.40)	< 0.001	< 0.001

Model 1 is unadjusted.

Model 2 is adjusted for age, gender, race, marital status, education and family income to poverty ratio .

Model 3 is adjusted for Model 2, body mass index, smoking status, alcohol drinks, hypertension, diabetes, congestive heart failure, stroke, total cholesterol and serum bicarbonate.

^a^*CI* = Confidence interval

**Fig. 2:**
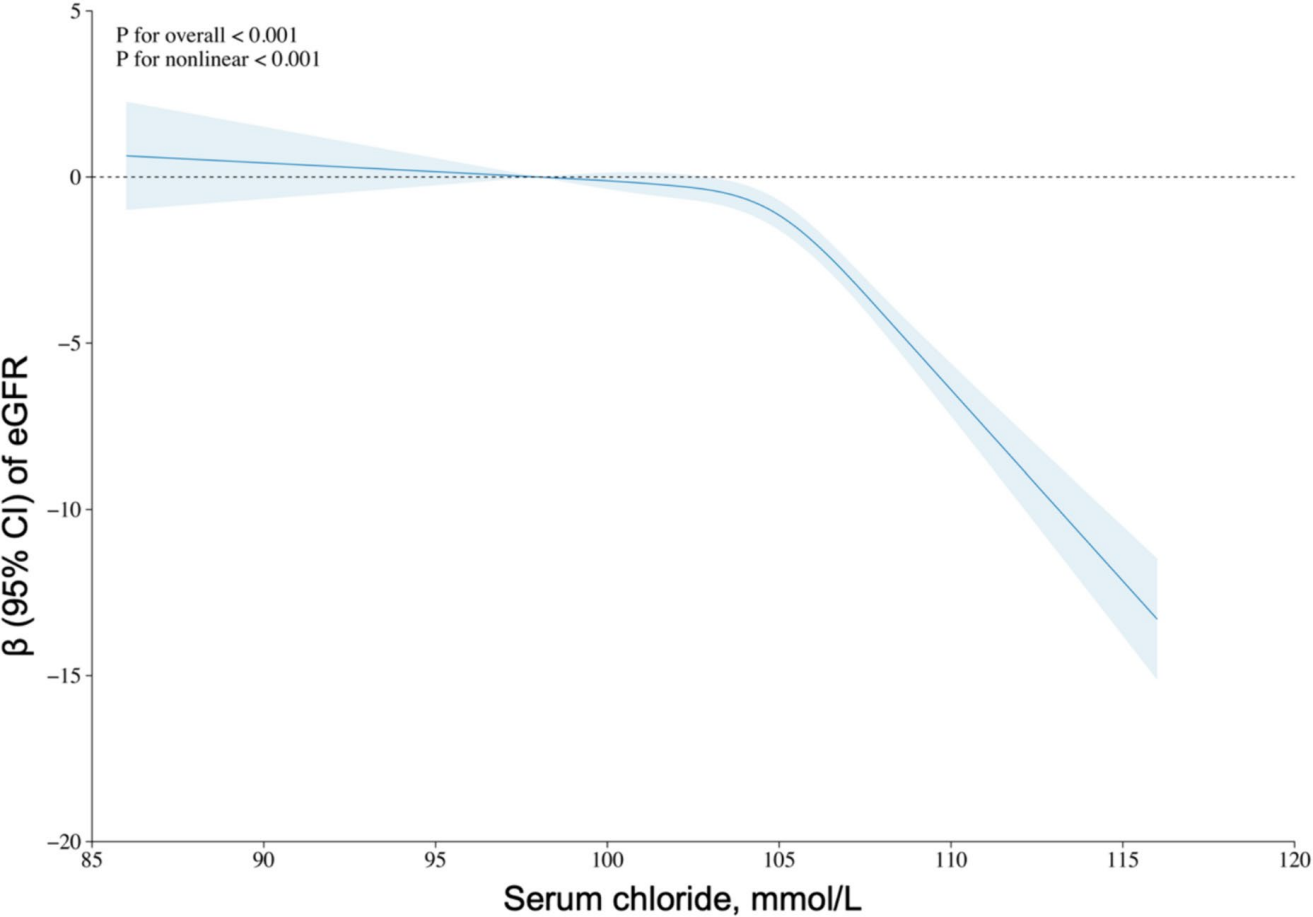
Restricted cubic spline (RCS) analysis with multivariate-adjusted associations between serum chloride levels and eGFR in adults. Models adjusted for age, gender, race, marital status, education level, family income to poverty ratio, body mass index, smoking status, alcohol intake, hypertension, diabetes, congestive heart failure, stroke, total cholesterol and serum bicarbonate. *eGFR* estimated glomerular filtration rate

To investigate the association between serum chloride levels and eGFR, we analyzed restricted cubic spline (RCS) plots for the comprehensively adjusted model. The outcomes of multivariate linear regression using RCS are presented in Fig. 2. The findings indicated a significant nonlinear association between serum chloride concentration and eGFR (*P* < 0.001 for nonlinear) in the RCS (*P* < 0.001 for overall).

### Stratified analyses

Among the subgroups defined by sex, family income to poverty ratio, BMI, hypertension, and diabetes, we observed a significant interaction between chloride plasma concentration and them with eGFR (all *P* < 0.001 for interaction). In the subgroup exhibiting hypertension and diabetes versus the control group (the first quartile), the β values for eGFR in the fourth quartile stood at −2.2 (95% CI −3.4, −1.1, *P* < 0.001) and −2.2 (95% CI −4.2, −0.14, *P* = 0.036), correspondingly. When comparing the subgroup without hypertension and diabetes to the reference group, the β coefficients for eGFR in the fourth quartile were − 2.4 (95% CI −3.5, −1.3, *P* < 0.001) and −2.3 (95% CI −3.3, −1.3, *P* < 0.001), respectively. In the analysis that partitioned subjects based on BMI into categories of less than 25, between 25 and 29.9, and 30 or greater kg/m^2^, compared with the reference group (the first quartile), the β of eGFR in the fourth quartile was −2.6 (95% CI −3.9, −1.3, *P* < 0.001), −1.8 (95% CI −2.8, −0.72, *P* = 0.001) and −2.6 (95% CI −3.8, −1.4, *P* < 0.001), respectively. Nonetheless, the analysis failed to reveal any statistically significant interactions between serum chloride levels and other strata variables about eGFR (Table 3).

**Table 3:** Associations between serum chloride levels and eGFR in various subgroups among individuals from NHANES 1999–2018

	Chloride plasma concentration, mmol/L							*P* value for interaction
	Quartile 1 (70–101.3)	Quartile 2 (101.4–103.4)		Quartile 3 (103.5–105)		Quartile 4 (105.1–120)		
Characteristic	β (95% CI^a^)	β (95% CI^a^)	*P* value	β (95% CI^a^)	*P* value	β (95% CI^a^)	*P* value	
Age, years								
≤ 39	Reference	−1.70 (−2.60, −0.74)	< 0.001	−1.80 (−2.90, −0.64)	0.002	−2.90 (−4.30, −1.50)	< 0.001	0.520
40–59	Reference	−1.50 (−2.50, −0.49)	0.004	−2.00 (−2.90, −1.10)	< 0.001	−2.60 (−3.90, −1.30)	< 0.001	
≥ 60	Reference	−0.96 (−2.00, 0.03)	0.058	−1.10 (−2.10, −0.14)	0.026	−1.80 (−3.10, −0.56)	0.005	
Gender								
Male	Reference	−1.50 (−2.30, −0.73)	< 0.001	−1.50 (−2.30, −0.74)	< 0.001	−1.80 (−2.90, −0.76)	< 0.001	< 0.001
Female	Reference	−1.80 (−2.50, −1.10)	< 0.001	−2.30 (−3.10, −1.60)	< 0.001	−2.90 (−4.00, −1.80)	< 0.001	
Educational level								
Less than 9th grade	Reference	−0.87 (−2.40, 0.65)	0.300	−1.30 (−2.70, 0.24)	0.100	−1.30 (−2.70, 0.09)	0.067	0.860
9–11th grade	Reference	−1.00 (−2.40, 0.29)	0.120	−2.00 (−3.40, −0.55)	0.007	−3.30 (−4.90, −1.60)	< 0.001	
High school grad/GED or equivalent	Reference	−1.90 (−3.10, −0.60)	0.004	−2.40 (−3.60, −1.30)	< 0.001	−2.90 (−4.40, −1.50)	< 0.001	
Some college or AA degree	Reference	−1.40 (−2.30, −0.43)	0.005	−1.30 (−2.30, −0.33)	0.010	−2.10 (−3.30, −0.77)	0.002	
College graduate or above	Reference	−2.30 (−3.40, −1.20)	< 0.001	−1.90 (−3.00, −0.76)	0.001	−1.80 (−3.20, −0.34)	0.016	
Family income to poverty ratio								
<1.0	Reference	−0.18 (−1.30, 0.93)	0.700	−0.87 (−2.10, 0.35)	0.200	−2.00 (−3.60, −0.37)	0.017	< 0.001
1.0–3.0	Reference	−1.50 (−2.30, −0.63)	< 0.001	−2.20 (−3.00, −1.50)	< 0.001	−2.90 (−3.90, −1.90)	< 0.001	
>3.0	Reference	−2.30 (−3.20, −1.50)	< 0.001	−1.80 (−2.70, −0.93)	< 0.001	−2.00 (−3.20, −0.71)	0.002	
Body Mass Index								
< 25.0	Reference	−2.50 (−3.40, −1.60)	< 0.001	−2.00 (−3.10, −0.91)	< 0.001	−2.60 (−3.90, −1.30)	< 0.001	< 0.001
25.0–29.9	Reference	−1.40 (−2.40, −0.42)	0.005	−1.80 (−2.70, −0.92)	< 0.001	−1.80 (−2.80, −0.72)	0.001	
≥ 30.0	Reference	−1.20 (−2.10, −0.35)	0.007	−1.70 (−2.70, −0.69)	0.001	−2.60 (−3.80, −1.40)	< 0.001	
Hypertension								
Yes	Reference	−1.50 (−2.30, −0.79)	< 0.001	−1.30 (−2.00, −0.53)	< 0.001	−2.20 (−3.40, −1.10)	< 0.001	< 0.001
No	Reference	−1.70 (−2.50, −0.90)	< 0.001	−2.30 (−3.20, −1.40)	< 0.001	−2.40 (−3.50, −1.30)	< 0.001	
Diabetes								
Yes	Reference	1.10 (−1.00, 3.10)	0.300	−0.06 (−1.80, 1.60)	>0.900	−2.20 (−4.20, −0.14)	0.036	< 0.001
No	Reference	−1.90 (−2.50, −1.30)	< 0.001	−2.00 (−2.70, −1.30)	< 0.001	−2.30 (−3.30, −1.30)	< 0.001	

Adjusted for age, gender, race, marital status, education and family income to poverty ratio, body mass index, smoking status, alcohol drinks, hypertension, diabetes, congestive heart failure, stroke, total cholesterol and serum bicarbonate. The strata variable was not included when stratifying by itself.

^*a*^*CI* Confidence interval

## Discussion

This cross-sectional study, which incorporated 49,008 participants from the NHANES from 1999 to 2018, demonstrated an inverse correlation between serum chloride levels and eGFR. Elevated levels of blood chloride have been found to correlate with diminished eGFR, even after accounting for confounding factors that could influence either blood chloride levels and/or eGFR. RCS analysis disclosed a nonlinear relationship between serum chloride concentration and eGFR within the general population. This is, to our knowledge, the first study examining the relationship between eGFR and chloride plasma concentration in the broader US community. Our research contributes to the increasing amount of data suggesting that chloride may play a part in human illness.

Previous studies have primarily concentrated on patients afflicted with cardiovascular diseases, including heart failure, hypertension, and pulmonary arterial hypertension. Hypochloremia is associated with an increased likelihood of cardiovascular deaths among people, regardless of the presence of known cardiovascular hazards and other electrolyte levels, like serum sodium [40]. Recent studies have indicated that, in the context of chronic heart failure, serum chloride levels may be a more robust predictor of patient outcomes compared to serum sodium levels. Studies conducted by BEST trial indicated a strong correlation with increased death risk due to hypochloremia, exhibiting a hazard ratio (HR) of 1.3 (95% CI 1.18–1.42; *P* < 0.001) corresponding to each decrease in serum chloride levels by standard deviation. The link persisted irrespective of serum sodium concentration, a condition that did not affect death risks considering serum chloride amounts [41]. In a prospective cohort study with 1673 participants revealed a link between reduced chloride concentration, quantified per standard deviation, and a heightened adjusted mortality risk, evidenced by a hazard ratio of 1.29 and a 95% CI between 1.12 and 1.49, holding statistical importance (*P* < 0.001). When chloride levels were incorporated into a multivariable model, they resulted in a net reclassification improvement of 10.4%, which was statistically significant (*P* = 0.03). In contrast, sodium levels did not demonstrate prognostic significance within the same model (*P* = 0.30) [29]. Similar findings have been observed in the TOPCAT trial [42], associating reduced serum chloride with heightened risk of hospitalization for heart failure, cardiovascular fatalities, and overall mortality. The study clarified that reducing serum chloride levels by 4 mmol/L correlates with a heightened risk of death from various reasons, including cardiovascular incidents. Hazard ratios were determined to be 1.29 (95% confidence interval [CI] 1.02–1.62; *P* = 0.04) for all-cause mortality and 1.51 (95% CI 1.11–2.06; *P* < 0.008) for cardiovascular mortality, correspondingly. There is a connection between hypochloremia and negative results in individuals suffering from acute decompensated heart failure. A review of more than 1300 heart failure patients, sorted by admission serum chloride and sodium levels, showed a link between hypochloremia and declining survival rates over a 3-year monitoring span. Conversely, hyponatremia did not significantly affect survival rates if chloride levels were maintained within the normal range [28]. Over a 35-year period, McCallum and team, evaluating 12,968 hypertensive patients in their comprehensive study, found a 20% increased risk of death among those in the bottom quintile for serum chloride levels (less than 100 mEq/L), covering all causes including both cardiovascular and non-cardiovascular, compared to those treated with a higher serum chloride counterparts. Gradually, every 1 mEq/L increase in serum chloride correlated with a reduction of 1.5% in the risk of all-cause death (HR, 0.985; 95% CI 0.98–0.99), a relationship that remained even after accounting for initial variables such as serum sodium, potassium, and bicarbonate levels [32]. A research with 277 individuals suffering from idiopathic or inheritable PAH inheritable individuals with serum chloride concentration of 100 mM/L or less at 6 months showed a higher death rate. After accounting for factors such as age, gender, pulmonary vascular resistance, the use of prostacyclin or diuretics, and serum creatinine and sodium levels at 6 months, this link stayed statistically meaningful, resulting in a hazard ratio of 1.83 and a 95% confidence interval between 1.11 and 3.00 [30].

The profound interconnection between cardiac and renal physiology is paramount and warrants emphasis. The kidney is where chloride is mainly excreted. Historically, investigative efforts examining plasma chloride levels and renal function have predominantly centered on the context of AKI. Studies investigating the connection between blood chloride levels and eGFR are very rare. Yunos et al. [43] conducted a pre-post clinical experiment with more than 750 individuals to assess the effects of intravenous chloride levels on kidney health. During the period when a chloride-restricted intravenous fluid strategy was implemented, there was a notable decline of 50% in AKI occurrences as per the RIFLE criteria, along with a reduced use of renal replacement therapy (RRT), dropping from 10% in the control stage to 6.3% during the entire intervention period. In a retrospective study involving 250 adult participants within a multidisciplinary academic intensive care unit (ICU), 57% of participants developed hyperchloremia within the first 48 h post-ICU admission. Elevated chloride levels at 48 h were found to be a significant forecast of AKI, with an odds ratio (OR) of 6.44 (95% CI 2.95–14.10), and of increased mortality, OR of 2.46 (95% CI 1.22–4.94) on univariate studies. These correlations remained consistent upon multivariate analysis [44].

The study synthesized findings on the relationship between serum chloride concentration and eGFR. A negative association was delineated, with eGFR being discernibly reduced in subjects exhibiting elevated serum chloride levels. The determination of whether the correlation between chloride fluctuations and long-term eGFR alterations has a causal underpinning or significant clinical implications remains to be established through additional research and corroboration. Nonetheless, there exist potential physiological pathways that could underlie and elucidate the connections observed in these results. The chloride ion is essential to physiological processes, including the regulation of both extracellular and intracellular volume, as well as maintaining acid–base homeostasis. Research conducted on experimental animals has shown that it is the plasma chloride, not sodium, that results in denervated canine kidneys having less renal blood flow [26]. Studies have demonstrated that both Angiotensin II (ANG II) [45] and endothelin [46] contribute to the enhancement of chloride ion conductance, alongside mediating vasoconstriction in the afferent arteriole. Furthermore, the gradient between extracellular and intracellular chloride has been demonstrated to be critical in determining the responsiveness of the afferent arterioles. Low levels of chloride in the macula densa lead to an increase in the production of renin and local prostaglandins when there is a decrease in perfusion pressure. These mechanisms work through afferent vasodilation and efferent vasoconstriction to maintain GFR. An excess of chloride in the body, whether from normal saline or due to hyperchloremia, enhances the transport of chloride towards the macula densa in the distal region. This triggers a reduction in GFR through afferent vasoconstriction, a process facilitated by thromboxane and adenosine. Concurrently, there is a diminished response in efferent vasoconstriction attributed to decreased levels of renin and angiotensin II [47].

An alternative hypothesis accounting for the noted correlation could relate to metabolic acidosis, considering that elevated serum chloride concentration frequently occur alongside metabolic acidosis, a condition linked to an escalated risk of CKD advancement [48, 49]. This occurrence is explicable by the fact that metabolic acidosis elevates the process of ammoniagenesis, consequently amplifying the excretion of acid. Despite the overall decrease in ammonium excretion observed as CKD progresses, the generation of ammonia on a per-nephron basis experiences an upsurge, contributing to the sustenance of augmented acid secretion in the context of nephronic impairment [50, 51]. The adaptive response proves detrimental to the survival of nephrons, as it augments ammoniagenesis, leading to an elevated local ammonia concentration around the nephron. This, in turn, can initiate the activation of complement C3 and C5b-9, as well as the alternative complement cascade. Detrimental outcomes of this maladaptive activation of the complement system include the overproduction of inflammatory and fibrogenic factors. These mediators are implicated in exacerbating proteinuria, inflammatory responses, and fibrotic processes, thereby aggravating tubulointerstitial damage and the progression of CKD [52].

The present research possesses multiple noteworthy advantages that enhance its validity and reliability. This investigation encompassed a nationally representative sample, thereby enabling the extrapolation of the results to the broader United States populace. Furthermore, the study leveraged the comprehensive and robust data acquisition inherent in the NHANES to mitigate the influence of confounding variables encompassing demographic characteristics, socioeconomic status, dietary habits, lifestyle choices, health conditions, and familial chronic disease histories. Third, the employment of skilled personnel adhering to uniform procedures for the ascertainment of anthropometric measurements, such as height and weight, as well as the systematic gathering of laboratory and interview-based information, serves to augment the precision and veracity of the resultant dataset. Last but not least, analyses of subgroups were conducted to evaluate the strength of the association between serum chloride levels and eGFR across diverse cohorts.

Notwithstanding its merits, this investigation acknowledges multiple potential constraints. First, because exposures and outcomes are recorded simultaneously, cross-sectional studies, like the one we used, have some limitations. Therefore, the only use for our data is to evaluate associations. They cannot even be utilized to evaluate the temporal or directional nature of the observed relationships, let alone establish causality. Second, the prospect remains that certain confounding variables may not have been sufficiently addressed, potentially permitting the persistence of residual confounding elements and unidentified confounding factors that cannot be unequivocally ruled out. In addition, the evaluation of renal function was limited to a singular time point, disregarding anomalies that may occur under physiological conditions or those that emerge from AKI. Lastly, the restricted accessibility of extensive drug-related data in the NHANES database hindered our ability to assess the influence of medications, such as dialysis treatment and the utilization of diuretic medication, on our results. It is anticipated that future research initiatives will include additional covariates to enhance the validity of our findings.

## Conclusion

The findings of this study, which focused on Americans adult, suggest that there may be a connection between higher plasma chloride levels and a higher likelihood of decreased eGFR. These findings underscore the critical role of monitoring chloride plasma concentration as a potential indicator for identifying individuals at risk of developing CKD. Nevertheless, to solidify these observations, further large-scale longitudinal investigations are desperately needed to validate the findings of this study.

## Data Availability

Researchers and data users from all over the world can access the survey data on the internet (www.cdc.gov/nchs/nhanes/).
